# Pain, Shame, and Power: An Autoethnographic Exploration of Chronic Pain

**DOI:** 10.1177/10497323241289805

**Published:** 2024-11-20

**Authors:** Katharine Wakelin

**Affiliations:** 1 School of Education, 6123 University of Nottingham, Nottingham, UK

**Keywords:** embodiment, chronic pain, autoethnography

## Abstract

In this autoethnographic study, I explore my lived experience of a chronic pain condition, the difficulty in writing about embodied experience, and the links between pain, shame, and power. Neglecting neither the complex emotional world of the individual nor the embedded cultural and social themes that continuously impact on the individual, at its best, autoethnography bridges the divide between personal writing and social influences. In this paper, I aim to combine my lived experience of a pain condition without any apparent biological cause, to the wider issue of how we conceive and attend to embodied experience, shame, and power in qualitative health research. The implications from the study include personal emancipation, challenging the mind/body split, and emphasizing the interconnections between emotion and embodied experience, and the need for a pluralistic approach to treatment. The autoethnographic approach aims to embrace situated subjectivity and to include the experience of being a pain sufferer in the research community.

## Introduction

Chronic pain is a lived experience I had for over a decade and an experience I viscerally wanted to escape from and deny. While my experience of pain preoccupied me in many ways, including searching for treatments, trying to understand the meaning of my situation, handling the side effects of medication, and exploring the emotional fallout of my experience, I spoke little about the pain apart from with professionals. I came to understand that this secrecy and the accompanying shame I felt were key components of having a chronic pain condition. It was as if I had moved into a lived experience that I could not explain or express to others. Trying to make sense of this shame along with my interest in how mind/body conditions develop and the relationship between pain and emotion prompted me to write this autoethnographic account of my experience. This has been made possible by a diminution of my symptoms, stopping taking medication, and recovering a sense of nearly health, a return to the land of the “normal,” which has emboldened me to try and put into words the experience of living with chronic pain.

A note on language: I have struggled when describing and now writing about pain to put the experience into words both in the sense that I found it a hard topic to talk about and that I grappled with finding the appropriate language. “My” pain sounds proprietorial, and possibly even proud. At times, I have adopted that language to explore pain as something I am creating, experimenting with *owning* the experience: I am creating the pain in my head. Clearly, this language risks self-blaming and self-shaming and can increase the sense of despair and isolation with pain. The alternative is to think of the pain as something *other* than me, an alien invader, that has taken over my head and is tormenting me. This approach risks being infantilizing and disempowering, something is happening to me that I have no influence over. Neither is entirely satisfactory, and it can be difficult to navigate the mind/body split that is inherent in much of our language around pain. At the same time, putting language to the experience is a valuable process that helps promote self-agency. Another way to manage the difficulty of articulating pain experiences is through using images of pain, which may help pain sufferers communicate their experience when words seem inadequate or elusive (Padfield et al., 2018). I have included two images I made to explore aspects of the pain experience that were hard to put into words.

## My Own Experience of Pain and Shame

When I was 37, with two small children and very little time for myself, sleep-deprived, and with an emotional sense of feeling trapped, experiencing both a lack of support and a lack of a sense of self-agency, I developed a series of sinus infections that were treated with antibiotics. Each time I recovered, until on one occasion the antibiotics did not work, and the pain stayed permanently, although the infection itself cleared. That time I felt a particularly strong sense of loneliness, of not being understood. I felt despair and fear. I had not gone back to work, my time was dedicated to my children, and unlike with my first child, I did not have a circle of friends with whom to share the experience. I also had a life-long difficulty in asking others for help, a belief that I needed to be self-sufficient and “tough” and a sense of shame over my vulnerability and difficulty in coping with the situation. My longing for someone else to take care of me and sort out the pain became subterranean and surrounded by fear, shame, and despair.

The pain was in my head, at the front of my face on either side of my nose and above my eyebrows and more deeply into my head; it was continuous and on both sides. The sensation was a dull, heavy ache. I would wake up with the pain, and it would gradually get worse as the day went on. There could be sharp throbbing tones to add to the ache as it got worse. Because it was in my head, it affected many of my senses and I felt like it was striking in a place where I found it particularly hard to evade or ignore the pain. Over the course of the day, it would come to dominate my experience. Without medication, the pain was unbearable, and I would retreat to lie down in a dark room. Reading or looking at a screen was impossible. With medication, I could undertake most activities, though the pain was not completely absent, and the medications had some side effects such as fatigue that were also challenging; the ache would return as the day wore on.

I also had an emotional response to being in pain. I can remember waking up in the spare room at my parents’ house and looking at the walls and feeling a falling sensation inside, both a vertiginous sense of being at a great height and falling, and a closing in and collapsing sensation. I was not thinking, or at least not thinking coherent thoughts, instead I felt a sense of panic and helplessness and a conviction—not really a thought, more a belief, part of what psychoanalyst Christopher Bollas has termed an “unthought known” (Bollas, 1987)—that no one could help me. I felt locked into a sensation of pain that was by its nature, invisible to others.

At that time, I was already a psychotherapist, although I was not working, and I was in weekly therapy with a trusted therapist I had been seeing for several years. Nevertheless, despite that support, the overall situation I was in was not conducive to healing for a few key reasons including a lack of support in the environment, and the attitudes and beliefs I brought to the situation from my own upbringing, particularly my attitude to illness. The gap between what I was feeling and what I felt I *should* be feeling was fertile ground for feelings of shame and vulnerability. Shame can be profound including a sense of not being good enough, deficient, or unacceptable, but also with feeling like the self has no right to exist: “shame is the experience of one’s felt sense of self disintegrating” (DeYoung, 2015, p. 18).

Much has been written about the link between chronic illness and shame (Murphy et al., 2022; Thamm et al., 2024) and the sense of deficiency that attaches to illness and pain in a society that prizes health and normality. In Charmaz’s study of chronic illness, she finds “living with a serious illness … means overcoming stigmatizing judgements, intrusive questions, and feelings of diminished worth” (Charmaz, 1991, p. 2). In my case, this was intimately linked to a history of family shame around illness or pain, so that in my family of origin, talking about illness or pain was prohibited and disapproved of as attention-seeking or indulgent. This meant that as soon as I felt ill or in pain, I also felt ashamed and adopted an on-going defense of attacking my self, avoiding the pain, or withdrawing from others (Nathanson, 1992).

Not only did I need to manage the shame of having a chronic condition, but also the lack of perceived legitimacy to my lived experience. The experience of pain, not necessarily linked to an organic cause (as the sinus infections appeared to have cleared), was a situation in which even the dominant experience of being in pain could be questioned: doctors were telling me the infection was gone, I “should” no longer be feeling pain.

I am sitting in the waiting room of a consultant, hoping for a possible diagnosis and treatment. I feel outside of myself, overwrought and stretched thin. My head is aching with a drilling pain in the temples and forehead, the room is hot and oppressive. I find myself staring at the bare, cracked linoleum and I have a fleeting image of myself lying on it, screaming and hitting out in a toddler frenzy. Instead, I dutifully wait my turn. I am called in, and I pause uncertainly on the threshold of the room. The distracted consultant is frowning at the notes on his desk. Almost before I have sat down, he tells me I have never had a sinus infection, that is not an explanation for the pain. He seems pleased I am not his problem: “it’s impossible the pain is sinus related, you would have scars and you don’t have any.” Panic rises in my throat; I sense a gap between myself and the world. I want to shout out I’m a mother, I have a good job, I cannot bear the nakedness of my own vulnerability. But I cannot find my voice. I walk out of the hospital. A couple are seated on a bench eating ice cream, the sun lends a holiday atmosphere to the day, yet I feel alienated from this normality. I drag myself to the bus stop. I have let myself down again. I am clogged up with despair and frustration. I feel as if the doctor could as well have punched me in the stomach, spat in my face, the effect of his words is devastating.

What was happening to me was invisible, I could not prove or show the pain that I was feeling and that locked me into greater isolation and shame (Birk, 2013). Having a language and a framework for my experience was something I longed for and set out to understand. As Probyn (2005, p. 60) explains, shame is both embodied in individual emotional experience and sociological “as it hits us physiologically, shame triggers reactions in individual psyches and at a broader social level.” My own history of stoicism and avoidance of pain interacted with ideas both about women and more generally about society’s shame around illness, to add to a sense of myself as fragile and unworthy. Finding an order or pattern to my experience, and making sense of it, was an overwhelming drive at that time. The contingent, inexplicable nature of my pain experience, which felt chaotic and senseless, contributed to my sense of shame.

## Why Autoethnography?

As the name implies, autoethnography involves the combination of two components in a research method: self and society. Successful autoethnography links personal experience to large social themes although the emphasis may vary with both evocative, descriptive work and more analytical, thematic approaches (Chang, 2016; Ellis, 1999; Klingler, 2024). I aim to combine the two, locating descriptions of my embodied experiences in a more sociological context as well as hoping to “reveal the profane in the sacred processes of research” (Ellingson, 2006, p. 304). As this means combining the granular detail of lived experience with an understanding of the social context in which it occurs, it is an ideal approach for psychotherapists, who share an emphasis on reflexivity as a way of knowing using embodied experiencing, images, and associations as data (Ciotti, 2023; Hills, 2023).

Autoethnography provides a framework to link the personal to the political and sociological, and to turn what could be seen as disadvantages—the lack of neutrality and power—into advantages, by giving voice to an aspect of the experience that has been missing, that of the patient, who, while not neutral or empowered, has precious lived experience that can help us understand a complex and elusive phenomenon such as pain. In the evocative description of Ellis and Bochner (2000), autoethnography can “substitute the companionship of intimate detail for the loneliness of abstracted facts” (p. 744).

Neglecting neither the complex emotional world of the individual nor the embedded cultural and social themes that continuously impact on the individual, at its best, autoethnography bridges the divide between personal writing in psychotherapy and sociology. In the context of storytelling as an approach to illness, Frank (2013) writes that the “voice is embodied in a specific person, but it is equally social” (p. 18). A growing literature provides first-person accounts of living with pain and illness (Birk, 2013; Ciotti, 2023; Ettorre, 2005; Frank, 2013; Neville-Jan, 2003) and documenting pain experiences through qualitative research (Sheppard, 2020; Werner et al., 2004). An autoethnographic approach has been used movingly to consider the role of medical ethics in gaining informed consent in the context of breastfeeding (Klingler, 2024) and the impact of combining a professional and patient role, or researcher and patient role on the lived experience of pain (Ciotti, 2023; Klingler, 2024). Psychotherapists have adopted autoethnography to explore various embodied experiences of pain (Ciotti, 2023; Richards, 2023). I would like to add to the first-person narrative tradition, to interrogate the relationship between emotion and physical pain and to link my own experience to the broader literature on mind/body experiencing and self-reflective research.

Different taxonomies have been used to consider illness narratives that may or may not be autoethnographic. Richards (2008) identifies three: testimony, which details the individual struggle from lived experience; emancipatory, which aims to give a voice to the voiceless; and the destabilized narrative. The last tries to escape the illness as temporary disruption narrative (my own could fall into this category) where the narrator recovers and “normality” resumes, possibly with a moral or emotional story about how the individual overcame adversity. Frank (2000) destabilizes this narrative and resists the trite resolution of suffering and instead focuses on the complexity and uncertainty of illness.

## Exploring Ethics

I have used written diaries and a notebook of pictures and free drawing that I kept daily for a period as source materials along with my visceral memories of much of my emotional experience. Although many of my experiences involved others—my family, medical professionals, psychotherapists, and alternative practitioners—none will be mentioned in such a way that needs ethical approval (Ettorre, 2005). Nevertheless, as Ellis (2007) points out, ethics can have a much wider interpretation and may be procedural, situational, and relational. I have an ethical responsibility to be accountable in the way I portray others, and I have elected to give this paper to many of the people involved to check they feel comfortable with my version of the experience. None has asked for any changes, which while convenient may also reflect what Etherington (2007) notes as the importance of attending to the power dynamics in gaining ethical consent: do others feel a real ability to refuse or question the narrative being put forward, to challenge the narrator’s version. I hope that this was the case, but in the absence of any real disagreement over my version, I have no way of knowing. The choice I wrestled with most was whether to share the paper with my children, now 19 and 17, as I was concerned that they might find it difficult to read, perhaps both boring and too revealing. I reflected on this decision and discussed it with friends and colleagues, before deciding it was worth the risk, as they are both intimately involved in the experience, and I would be able to respond to any doubts or worries they had. A friend pointed out I was focusing on the disadvantages, without seeing the advantages of having two new perspectives. We had some thoughtful and frank conversations about my experience; both were clear that they in no way feel responsible, and I concluded that some of my misgivings had been a shame response, that on a visceral level I remain ashamed of having had a chronic pain condition, and the imperfections and vulnerabilities that go along with that. My children’s response of interest and concern helped allay some of those shameful fears and was an unanticipated benefit of the research process.

Another ethical consideration is the impact of sharing my own illness story and the ensuing loss of privacy. As Richards (2008) writes in the context of her kidney disease “in illness autoethnographies the story is particularly intimate and the telling of it can render the writer vulnerable” (p. 1718). I have reflected on this and am cautiously optimistic that both the process of writing about these experiences, and the act of sharing them, and reading others’ experience will lead to greater understanding and less isolation and shame. Despite my optimism, perils still abound: the risk of traumatization revisiting those times, the fear of reactivating the pain, not being able to do justice to the experience in words, and increasing my shame and vulnerability by being seen by others (Etherington, 2003). At its best, such autoethnographic explorations of illness allow the author to generate a new self and might serve an emancipatory function (Frank, 2013).

## The Mind/Body Split, Power, and Pain

The difficulty with language around pain mirrors and symbolizes our complicated relationship between the mind and the body. So much of our language supports a mind/body split in which we identify as our minds, as if we are carrying our bodies around. Frank (2013) movingly describes the difficulty of writing about embodied illness experiences: “we must speak for the body, and such speech is quickly frustrated: speech presents itself as being about the body rather than of it” (p. 2). No easy resolution to this dilemma presents itself, apart from trying to feel into and do justice to embodied experiences as researchers (Ellingson, 2006; Sharma et al., 2009).

Psychological approaches, including psychotherapy, often implicitly accept the mind/body split by neglecting embodied experiencing as belonging to the realm of medicine, and defining any crossover of psychological or emotional factors expressed as physical symptoms as *somatization* (or with Freud’s preferred term of conversion). The idea that a clear distinction between embodied experiencing with an organic explanation and those with a psychological explanation exists seems suspiciously binary (Leader & Corfield, 2007). A more nuanced understanding of distress acknowledges the complex and deep interconnections between mind and body. Hills (2023) “sought a term that did not separate off symptoms at all and settled on the term *embodied distress*” (p. 272), a looser term that can encourage practitioners (psychological and medical) to lean into lived experience rather than pre-emptively judging and separating the cause of the distress, a distinction which can aggravate the pain experience with the suggestion of invalidity and the dreaded implication it is “all in your head.”

The alternative to exploring embodied experience can be to treat symptoms as symbolic communications that need decoding (McDougall, 1989). In the psychoanalytic tradition, psychosomatic illness is often portrayed as a deficit—either a talking body or a speechless mind—either way, both “consider psychosomatic symptoms to be a result of psychic deficits” (Gubb, 2013, p. 135). Experience needs to be expressed and conceptualized in the verbal domain to move it from physical symptom to integrated experience. At one extreme, this can be a dualistic and unintegrated view of embodied experience as sensations in search of words and tends to be surrounded by language that implies deficiencies. At the same time, an important insight can be developed that our relational and developmental experience might be embodied in our adult experience.

Themes of power and powerlessness pervade many illness narratives (Ettorre, 2005; Neville-Jan, 2003; Thamm et al., 2024). Individualistic societies see health as reflecting individual morality and virtue, while conversely illness and pain reflect personal failure and lack of self-care (see, for instance, attitudes to weight, disability, and smoking in health discourses). Any chronic pain sufferer will come up against themes of power in the key doctor/patient relationship, as well as becoming aware of specific themes of power according to groups they may belong to, in my case being a woman and being educated, privileged, and white. Writing an autoethnography means reclaiming a sense of authorship over my own story, putting the words that so often eluded me to my own experience: “I might be able to escape from a narrative I did not write” Richards (2008, p. 1721). In part, this is a reaction and a rebuttal to the repeated experience of powerlessness that comes with illness. Much of my energy went into trying to fit my lived experience into a medical category that could access treatment for me and legitimize and validate my experience and alleviate the pain through treatment. Many write about this powerlessness in various illness contexts (Ciotti, 2023; Frank, 2013; Klingler, 2024) and it may be particularly pernicious with chronic pain, an experience that is both visceral and undeniable for the sufferer and hard to understand, identify, and treat for medical professionals (O’Sullivan, 2015).

The claiming of a first-person narrative of illness or pain is a challenge to the dominance of third-party accounts (Frank, 2013; Richards, 2008) and is an evolving field in health research (Ciotti, 2023; Klingler, 2024). This sense of being side-lined, and lived experience not being a sufficiently high-status way of *knowing* about something, is part of the experience of chronic pain and illness. While medical professionals know more about the cause and treatment of illness, and this knowledge is vital and valuable, the power imbalance in the doctor/patient relationship can negate or silence the voice of the patient both in the consulting room and outside. The insider voice articulating the messy embodied experience of being in pain has in the past been perceived as lacking both credibility and generalizability by others and by the self, making it doubly hard to be heard; we are both censored and silence ourselves (Klingler, 2024; Sheppard, 2020).

Many chronic pain sufferers are seen as attention-seeking or neurotic and many of them are women. Despite my advantages of presenting as an educated, privileged white woman, I was frequently patronized and dismissed by medical professionals (both men and women). At times I found it difficult to give voice to my experience in the face of professional indifference or hostility and instead minimized and belittled my lived experience before the medical professionals could or tried to demonstrate my credentials as a “worthy” patient, one who was working hard toward her own recovery and so deserving of the doctor’s time and patience (Wilbers, 2015). This gets to the heart of what Birk (2013) describes as the necessity of chronic pain sufferers to perform their pain, for others to understand it, in the absence of visible signs of pain. This makes it hard for the pain sufferer to maintain credibility in the face of skepticism, the need to perform pain that can then be experienced as inauthentic, along with, in my case, a lack of internal legitimacy of my pain status and a relational belief that my health needs would be belittled and ignored (DeYoung, 2015).

I search for answers in places that have brought me solace before, in books. So many books on pain, books on healing, books on psychosomatic illnesses. I visit alternative health practitioners, exhaustingly alert for new recommendations. I meditate, I extend my yoga practice. I am steadfastly active and committed to my own recovery with my habitual self-sufficiency. I bring my longing to each new encounter: this will be it; this will be the answer.

After the failure to get a diagnosis or treatment from the other consultant, after many delays, I am now seeing a neurologist. He’s young, relaxed, and he puts down the notes and looks me in the eye. It feels like he is less rushed, he takes a full history of what has happened to me from the beginning. Encouraged, I take the risk and haltingly recount my experience with the other consultant. He suggests that arriving at a diagnosis with such certainty is both medically and philosophically suspect: “it’s always possible you do not scar easily.” That feels like a balm to my pain, I want to lie my head down on his messy desk and cry I am so grateful to be understood. My head is spinning, and I feel a wave of relief inside, my pain lightens. The room feels suffused with soft light; I cannot find the words to tell this man how grateful I am. Here in the scruffy, institutionalized drabness of the NHS, with its discarded coffee cups and unstable towers of files, I have found someone who takes me seriously and can understand my experience. I dread this consultation ending. I’m tired of soldiering on alone. I feel a sob rising in my throat but do my best to choke it down. I need to be the good patient. I come out of the consultation feeling lighter, taller, my breath slower, and my heart pounding with excitement and relief.

Attitudes toward people in pain are gendered, with some evidence that women are asked fewer questions in consultations with doctors and sent for fewer diagnostic tests (Adams et al., 2008; Arber et al., 2004; Raine, 2000). This is reflected in qualitative research on health care, which has noted the way women’s pain is often dismissed or trivialized (Birk, 2013; Klingler, 2024) and women are concerned not to conform to the stereotype of the complaining woman in medical situations (Werner et al., 2004). In her wide-ranging meta-synthesis of gendered disenfranchising talk in health treatment, Hintz (2023) notes that “such talk functions to discredit their concerns, silence them, and reduce their experiences of illness to stereotypes” (p. 2511). This leads female patients to question the validity of their own experience, doubt their own credibility, and disengage from health services. This sustained feeling of powerlessness as a patient can lead to a change in the experience of self with the resultant development of an illness identity. Ettorre (2005) writes about both the joy and the difficulty of this process: “suffering and grappling with the intricate, interior language of wounding, despair and moral pain as well as the victory of living an illness” (p. 543). She suggests adopting the feminist position of “monadic flexibility” where the individual resists being typecast into a fixed social code. While I find this an uplifting view, refusing to be identified by the ready-made cliches around being a middle-aged woman in pain, I also found it enormously hard to achieve while I had the frequent experience of being a patient. It was only as I could claim more of my non-patient self that I managed to mend some of the dents to my sense of self rather than feeling like a supplicant in the health system, grateful for any half-way humane treatment.

## What Is Pain and What Is the Meaning of Pain?

My initial understanding of pain, probably the common understanding, is that pain is the product of an organic cause and is there to warn us of potential or actual biological damage, helping us to act and alleviate the pain. One of the unintuitive and radical ideas that I came across is that pain can be “real” in the sense that it hurts in the same way as biological damage, but it does not come from a physical cause. Rather, the brain, after repeated pain experiences, becomes specialized in pain and keeps giving the pain message long after it serves any useful behavioral purpose (Doidge, 2015; O’Sullivan, 2015). This type of pain is essentially a mistaken, hopeless signal, a ghost in the body, real to the experiencer, but without any biological meaning or purpose. This conceptualization partly contrasts to the psychoanalytic one (Gubb, 2013), which gives meaning to somatic symptoms. With this understanding, there may be no underlying psychic difficulty that gave rise to the chronic pain but rather a physical experience that the nervous system is pre-disposed to generate. And both can be true: symptoms may have meaning and can be seen as faulty wiring. I hesitate to use this metaphor as I partly dislike mechanical/electronic metaphors that seem reductionist, and the language we use around pain is itself revelatory about how we conceive pain (Hintz, 2023; Semino, 2023). Nevertheless, intuitively it captures the idea of biology and brain structure playing a part in pain. I developed a chronic condition partly because I was repeatedly getting infections, with some emotional causes to do with my own early life experience and the situation I was in, and this became chronic partly because of the way our nervous systems can become specialized in pain. This is an important point, as it means the treatment required is neither wholly physical nor wholly psychic. Instead, we need an integrated approach to treatment that addresses both the emotional and physical aspects of the experience.

This understanding took a long time to adjust to, partly because it can come with feelings of being a fraud, worry about not being taken seriously, and the lack of legitimacy of accepting “it’s all in my head.” It is also hard as the pain is a constant signal of potential harm and it feels frightening not to listen to that. Despite this new understanding, I experienced constant doubts: might I have a serious illness I’m not investigating, what if this is a neurotic symptom and therefore not “real.” This new-found understanding was accompanied by much emotional confusion over what to believe and the difficult process of faith around illness—who is credible and who can be believed both as a patient and in assessing potential treatments. Many people are selling snake oil and promising miracle cures to get rid of your pain, and the process of putting your faith and belief in someone can lead you on a rollercoaster of hope and crushing disappointment. At times, it felt easier to remain detached and not try, to stick with what you know, rather than risk being disappointed. I also found it hard to give up on a story I had told myself about my experience and adapt to a new understanding.

The body/mind separation dogs much of our language and understanding and makes treatment and meaning making complicated. Meaning making because we tend to think that physical sensations must have physical origins and it is hard to challenge this internally. Treatment because these tend to be either physical—doctor, osteopath, acupuncture, etc—or emotional—psychotherapist, EMDR (eye movement and desensitization and reprocessing therapy), and meditation—and though no one consciously wants to promote the mind/body split, an inevitable separation does exist when the opposite, integration, is what is really required. The psychotherapy literature with its language of somatization (McDougall, 1989) may unintentionally add to this with the idea that talking cures and emotion are the “correct” way of communicating rather than embodied experiencing. Some writers explore the personal meaning of illness including a spiritual or deeper meaning they may find in the experience (Ettorre, 2005; Neville-Jan, 2003). Nevertheless, mind/body issues are characterized by binary thinking that implies symptoms without a clear organic cause are imaginary, with the associated stigma and lack of understanding that goes with that characterization. This characterization also leads to misdiagnosis and a lack of integrated care with patients either being directed down a physical or mental health treatment track.

We also know from research that “stress”—emotional, physical, and social—has a long-lasting impact on health (O’Conner et al., 2021). We might naively assume a separation, in the sense that emotional stress is communicated emotionally and physical stress physically. Though this seems intuitive, it is only so because of the deep schisms around mind and body. Emotions are expressed through the body as well as through thoughts and feelings (which are themselves embodied) and physical stress including illness and injury impact on emotions and thoughts. Because the depth and complexity of these interactions are often overlooked, we can miss how one impacts the other. Language like “somatization” can be a terrible over-simplification, or a belief that expressing feelings through embodied experiencing is somehow “wrong” as if the wires have got crossed, rather than realizing it is an integral part of human experiencing: of course, we express suffering through our embodied experiencing.

**Figure 1.**
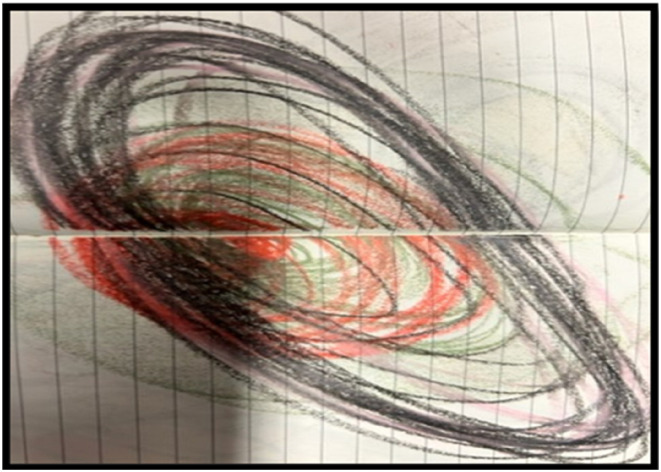
An early picture.

**Figure 2.**
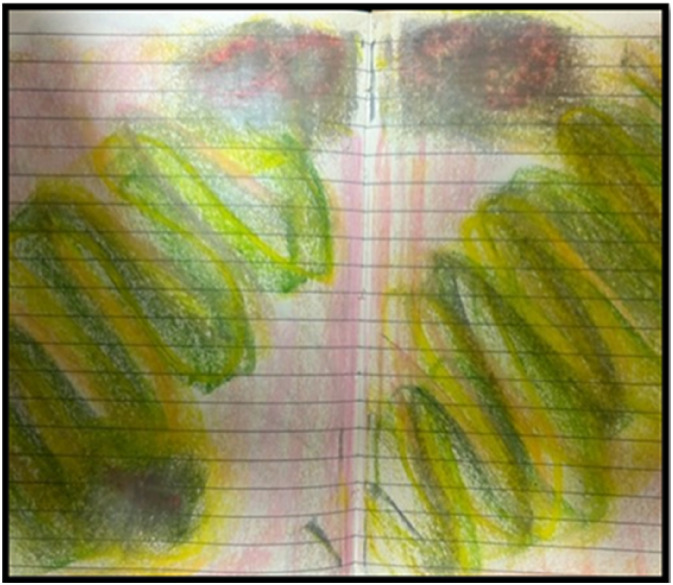
A later picture.

One of the emotional challenges of being in pain was that it was hard to claim my own agency in an area where others, in particular medical professionals, know more. It is tempting to give up power to the experts, but this keeps the feelings of powerlessness going. Building up a sense that I could do something about my experience through exercises, applying neuroscience’s evolving understanding of brain plasticity to pain management (Doidge, 2015) was a change of mindset into something more active and empowered. These exercises involved visualizing the pain, for instance, as parts of your brain on fire, and going round and extinguishing the fires one by one. The idea is to harness all your senses when you do this, to include a smell that can be associated with this (I used peppermint oil), to draw or paint the pain, to try and feel in your body the impact of extinguishing the pain as you visualize it. I used thick, paint crayons to draw the pain points in red, and then slowly covered over them in layers of other crayons—blue, yellow, and green—while relaxing and imagining the pain reducing in my mind. I have included two images here (see Figure 1 from early on in this process and then Figure 2 from over a year later), despite being tempted to omit them, a process akin to the disembodiment of much research that Ellingson (2006) evocatively terms “deceptively tidy accounts of research” (p. 301). The exercises require a lot of repetition, as you are trying to make new neural pathways to counteract those that conduct the pain and can be repeated many times in the day. The increase in a sense of agency then has a large emotional repercussion: I felt less helpless and had more choice around what was happening to me.

Increasing my own sense of agency also involved challenging emotional processes. The first was that I needed to give up on the fantasy of the omnipotent other who would make me better. I stubbornly held on to this, and it was a long, hard road to realize that I needed to be more active, with support of others, rather than waiting for the longed-for other to come and save me. An attachment theory approach to psychosomatic illness (Gubb, 2013) would link this to development deficits, with mis-attuned or neglectful early care, leading to an idealized longing for an attuned other. At other times of emotional hardship, I had imagined an all-seeing, all-caring other who would understand without explanation and see exactly what I needed. While this was a valuable imaginative aid in the past, in the circumstances I now found myself in, this imagined other substituted the hard conversations I needed to have with real people and articulating and acknowledging my needs and vulnerabilities in actual situations with health professionals. Over time, this combination of creative exercises and emotional work could be seen in my daily pictures (see Figure 2). The pictures also expressed something that could not always be put into words, and perhaps did not need to be.

## Exploring Treatment

In my case, no single treatment was the solution, it was a combination of approaches, and a slow accumulation of benefits over time that helped ease my pain. I had an experimental approach, trying many things in different combinations and often more than once and with different people. This makes it hard to predict what would work for a single person. As a relational approach would suggest (DeYoung, 2015), the treatment itself may not be the answer; the person delivering the treatment is also crucial. Most things that worked didn’t work the first time or didn’t work with some practitioners. The person seemed to be as important as what they do. This may be a combination of different levels of competency and mastery of their approach, the relationship with me, how I felt about the practitioner, how much trust I had, and that hard to control variable timing.

As my pain condition lasted a long time, many of the treatments had small cumulative effects and it is hard to draw out which were most effective. Those that worked best seemed to integrate embodied experience and emotion, either directly or because I did them at the same time. Craniosacral manipulation, which I had at the same time as EMDR, seemed to be effective together (and this was my second time doing both, neither was very effective first time). Craniosacral therapy is a subtle massage technique that can be used in the treatment of headaches and neck pain, while EMDR is a NICE-recommended treatment for trauma and is based on experiencing a right/left pulse or eye movement while processing traumatic memories. I imagine the efficacy this time was a complex mixture of attending to embodied experience (EMDR), being cared for by someone I trusted (both practitioners), having space to express my feelings associated with illness (EMDR) and timing. Building on the breakthrough I had with those treatments, where the pain on one side of my head disappeared completely, I then started to see a practitioner specializing in therapy for pain relief. This process involved somatic experiencing, going back to early memories of pain and illness, and exploring my embodied response to those experiences in the hear-and-now, introducing new resources to existing memories in the present Sommer Anderson and Sherman (2013). Those new resources may be visual, for example, changing the room the memory occurs in, or relational, adding in support that may have been missing at the time, a person you now know who can support and help you in the memory. The focus on early memories is partly to do with how we make meaning from illness and pain, and what function it may serve in our internal world. The central point is that people with chronic pain have an emotional response to illness/pain that comes from the way pain and illness was treated in their family, and the way they were treated by early caregivers when ill (Gubb, 2013; Hills et al., 2018). In my case, this approach helped me realize the despair and fear that I feel as soon as I become ill/feel pain—a belief that I couldn’t be helped/healed along with all the ways I resist accepting help and the chronic shame that I experienced along with illness or pain (DeYoung, 2015).

## Diagnosis and Medication

I actively sought out a diagnosis and took medication for almost a decade, gradually tapering it off over a 2-year period. Diagnosis (*knowing through* in Greek) is a way to understand your experience, both for the medical practitioner and for the patient, and indeed for wider society. It provides an anchor for all involved and reduces the sense of contingency and chaos present with much illness (Frank, 2013). Not having a name for your experience or the vague and elusive “medically unexplained symptoms” (Creed, 2016) might imply a bogus or unreal quality to your suffering, the suggestion that it is “all in your head” and reflects an underlying neuroticism or lack of emotional self-regulation that has manifested in physical symptoms. Patients deemed to have psychogenic pain are often then stigmatized with the associations to mental illness (Birk, 2013). But what we now understand about pain conditions is that pain can exist without an organic cause, but it does not make the lived experience of pain any different from if it did. Phantom limb pain which may affect 40%–80% of amputees (Urits et al., 2019) is a vivid example of this, where pain continues despite the removal of the limb. Given this is becoming the predominant understanding of chronic pain conditions, is it even helpful to think of a distinction organic/psychogenic pain when the treatment is no different as all pain will have both an emotional and a physical component (Leader & Corfield, 2007).

In my own experience, my emotional response to pain, conditioned in my early upbringing, and in a wider culture that admires stoicism, certainly contributed to my developing a pain condition. In addition, the intense situational factors at work at the time when the pain developed to do with motherhood, loss of identity, and a lost sense of competence and social belonging, all contributed to my not being able to recover.

## Conclusion

The thread that runs through this paper is the way illness is accompanied by shame, perhaps recalling earlier conceptualizations of illness as a moral failing, reflecting a sickness of the soul. More recent incarnations of this deficit thinking see illness as revealing psychic failures, failures to put emotions adequately into words, with psychosomatic illness as a defense against fully knowing oneself. The upshot is the same, the ill person is in some way to blame for their suffering, and they are stigmatized and marginalized as no longer part of a healthy society. Without holding on explicitly to those models of illness, shame remains pervasive around illness, reflecting a perceived failure of the self to live up to society’s ideal of a healthy self. This may also be a way for society to ignore the ever-present threat of illness and death (Frank, 2013) by locating the threat in marginalized outsiders. The resulting disembodiment also permeates much qualitative research in which “we often detach ourselves from the knowledge we produce, and we deny our bodily vulnerability” (Ellingson, 2006, p. 308).

Five main implications emerge from this focus on the lived experience of pain. The first is as part of the emancipatory discourse outlined by Richards (2008), in which a conventional narrative of chronic pain is disrupted and some power for the pain sufferer is reclaimed through giving a voice to lived experience. Pain renders the sufferer voiceless and often invisible, as well as compromised and stigmatized. By trying to put words to the embodied emotional experience of pain, I can try to expand and deepen our understanding of living with pain. Second, a nuanced account helps to resist the mind/body split prevalent in much of our thinking, with the two reductive alternatives of the medicalization of distress and the psychologizing of pain. Both approaches negate embodied distress and aggravate pain through the sense of being misunderstood and dismissed. Third, and this is most relevant for health research, I am trying to embrace the situated subjectivity of research rather than being embarrassed by it and reaching for the neat certainties of objectivity. Fourth, evocative description invites all of us to access our compassion and understanding of the other and humanizes the discourse around both research and pain. Finally, researching my own pain democratizes the pain sufferer as one of the research community, the subject rather than being the object of study to be pitied and prodded.

While I prefer to take an integrated approach to pain and embodied experiencing, using Hills (2023) term of embodied distress rather than psychosomatic illness, I also do not want to neglect the important role of emotion, and how we feel about illness, along with developmental issues that may leave us pre-disposed to embodied suffering. An important distinction exists between *having* a body and *being* a body, and conventional medicine, psychotherapy, and qualitative research can focus on the former, by excluding embodied experiencing. As Ellingson (2006) notes, “language conventions make it difficult to include the body as the self rather than the wholly owned subsidiary of the self” (p. 302). The best practitioners both saw me as a person experiencing embodied distress and attended to all aspects of that distress, either explicitly through an integrated treatment approach or implicitly by treating me as a suffering other who could be understood and helped. Likewise, qualitative research in health can also make space for the suffering body of the researcher as well as the researched.
